# Duty of Care Governance: a conceptual framework for aligning health system scale with the clinician–patient relationship

**DOI:** 10.3389/fsysb.2026.1796465

**Published:** 2026-05-25

**Authors:** Lawrence Merle Nelson

**Affiliations:** Mary Elizabeth Conover Foundation, Inc., McLean, VA, United States

**Keywords:** accountability, clinical governance, clinician-patient relationship, health system governance, health systems design, Primary Ovarian Insufficiency, relational continuity, systems theory

## Abstract

Modern health systems possess advanced scientific and technological capabilities, yet they often struggle to preserve the ethical basis of clinical care: the clinician’s primary obligation to the patient. When systems prioritize efficiency and large-scale growth, centralized authority can create structural tensions that may weaken the patient–clinician relationship, widening the gap between decision-making and patient outcomes. Duty of Care Governance is proposed as a conceptual structural model intended to re-anchor institutional design around this obligation. The framework organizes health systems around the clinician–patient relationship as the primary site of accountability. System integrity is conceptualized as being maintained through four interrelated governance functions: Sensing (interpretation of Clinical Signals), Responsibility (identifiable clinician accountability), Escalation (access to additional expertise without transfer of decision authority), and Feedback (longitudinal reassessment of outcomes). The system operates as a coordinated governance structure linking Clinical Signals with institutional guidance. Legitimacy depends on collaborative design, in which patients and clinicians co-author governing structures such as an organizational charter. This approach is intended to reduce relational displacement and organizational inertia. The manuscript introduces a financial perspective: the requirements of Duty of Care Governance can be understood not as administrative overhead, but as strategic investments that mitigate long-term liabilities such as clinician burnout, system failure, and patient burden. By contrast, evidence suggests systems that preserve relational continuity generate measurable value through improved outcomes and sustained trust. By maintaining structural discipline and anchoring authority at the site of care, the model is designed to preserve alignment between clinical decision-making and patient outcomes.

## Introduction

Modern health systems possess substantial scientific and technological capability, yet they often struggle to preserve the relational accountability that underpins the ethical legitimacy of clinical care. These challenges have been described in the clinical governance literature as failures of structured accountability and system-level quality assurance (Scally and Donaldson, 1998). The duty of care is historically grounded in professional norms and the autonomy of clinical practice (Freidson, 1970). The clinician’s duty of care to the patient is an obligation rooted in the professional relationship and requires action in the patient’s best interest (Starfield et al., 2005; Pereira Gray et al., 2018). Health systems function most reliably when governance preserves this duty of care as the organizing principle of institutional design.

Duty of Care Governance is proposed here as a conceptual structural model designed to protect and sustain this foundational obligation as systems scale, particularly by maintaining coherence across organizational levels (Nelson, 2026). This model is grounded in, and seeks to extend, prior empirical work demonstrating that relational continuity, sustained relationships between patients and clinicians, is associated with improved outcomes, reduced mortality, and more effective health system performance (Starfield et al., 2005; Pereira Gray et al., 2018).

The concept of duty of care is fundamentally a legal obligation. As defined, it is “a requirement that a person act toward others and the public with the watchfulness, attention, caution, and prudence that a reasonable person in the circumstances would use. If a person’s actions do not meet this standard of care, then the acts are considered negligent, and any damages resulting may be claimed in a lawsuit for negligence” (Law.com, 2026). Across professional domains, including law, engineering, and technology, the duty of care involves interpretive judgment and a commitment to clients’ or the public’s welfare. In the medical context, the distinction lies not in the presence of ethical obligation itself, but in the institutional conditions under which that obligation is exercised. Clinical care is characterized by high-stakes decision-making under uncertainty, longitudinal responsibility, and the integration of biological, experiential, and contextual information over time.

Within the clinician–patient relationship, essential clinical information is generated through the ongoing interpretation of biological data (e.g., laboratory markers), embodied patient experience (e.g., “the ouch”), and evolving treatment responses, reflecting a life-course perspective on health and illness (Kuh et al., 2003). For a system to remain responsive, governance mechanisms must allow this information to travel upward through organizations while preserving the interpretive context in which it was generated.

Conversely, when authority drifts away from the site of clinician accountability toward administrative management, continuity is disrupted, and the coherence of care is reduced. Continuity of care remains a core organizing principle of effective health systems (Starfield et al., 2005). This manuscript suggests that modern health care systems may experience breakdowns when authority drifts away from the human unit of care, and that this failure can be addressed by re-anchoring responsibility through Duty of Care Governance. To examine how this structural model relates to organizational expansion, it is first necessary to analyze how health systems scale.

## How health systems scale

Scaling in healthcare is not a neutral technical process; it reflects organizational choices about how authority, information, and responsibility are structured within health systems, particularly through the coordinating role of primary care (Starfield et al., 2005). Scaling is defined here as the multidimensional process of organizational expansion, technological deployment, and financial growth (Grace et al., 2025). To scale with integrity, systems require Scaling Literacy, the capacity to distinguish between Scaling Up (impacting laws and policy), Scaling Out (replicating models to increase reach), and Scaling Deep (transforming underlying cultures, hearts, and minds), a concept originating in the social innovation literature (Moore et al., 2015).

Across dominant models, including economies of scale, bureaucratic layering, and platform digitization, growth-centric approaches emphasize efficiency and expansion, with important implications for relational continuity (Grace et al., 2025). In these models, fragmentation can emerge from time constraints and competing demands within care delivery (Yarnall et al., 2003). Fragmented care refers to a structural condition in which the absence of sustained relational continuity leads to the separation of clinical information, decision-making, and Responsibility across providers, resulting in a loss of coherent, patient-centered understanding over time. By standardizing care to create “scalable assets” and optimize throughput, organizations can reduce visible unit costs. However, this centralization often succeeds at scaling “up” and “out” while failing to scale “deep,” which can weaken relational continuity and the clinician–patient bond (Starfield et al., 2005; Pereira Gray et al., 2018). The decisive question is whether scale mirrors the clinician–patient governance pattern or displaces it. Without governance discipline, expansion can contribute to fragmentation and reduced relational continuity, increasing the distance between clinical decision-making and patient outcomes (Yarnall et al., 2003; Pereira Gray et al., 2018). This tension between scale and relational accountability necessitates a governance structure explicitly designed to preserve the clinical core of care.

## Duty of Care Governance: conceptual framework

Duty of Care Governance addresses the challenge of institutional scaling by organizing health systems around the clinician–patient relationship as the primary site of accountability. In this model, the clinician–patient relationship is not merely an operational component, but the point at which professional responsibility, clinical judgment, and patient experience converge, consistent with evidence on the central role of primary care and continuity in care delivery (Starfield et al., 2005; Pereira Gray et al., 2018).

To preserve this core as systems expand, the framework is structured around four interrelated governance functions: Sensing (interpretation of Clinical Signals), Responsibility (identifiable clinician accountability), Escalation (access to additional expertise without transfer of decision authority), and Feedback (longitudinal reassessment of outcomes). Together, these functions maintain alignment between institutional structures and the relational core of care. This structure reflects principles described in high-reliability systems, including continuous sensing, structured escalation, and adaptive feedback (Weick and Sutcliffe, 2015).

**FIGURE 1:**
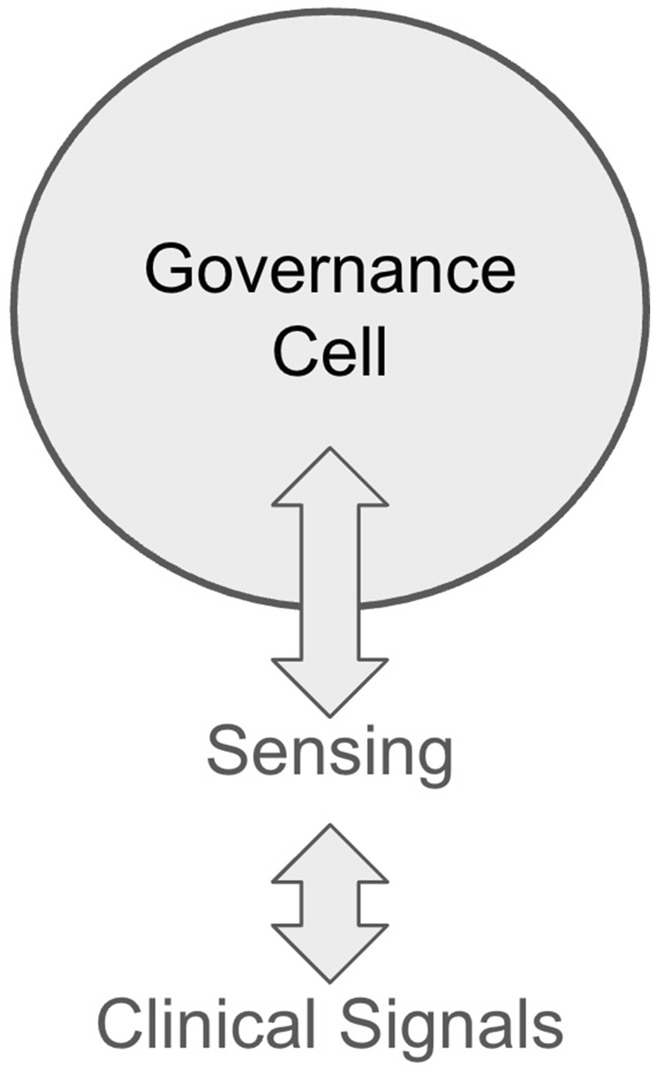
Interface between Clinical Signals, Sensing, and the Governance Cell. Legend: This figure depicts the clinical interface through which frontline information engages governance. Clinical Signals arising from patient care are bidirectionally linked with Sensing, representing the ongoing recognition and contextual interpretation of emerging concerns. Sensing is, in turn, bidirectionally coupled with the Governance Cell, which in this model is anchored in the clinician–patient relationship, with continuity as a defining feature, and serves as the locus for interpretation, accountability, and coordinated response. The bidirectional arrows indicate that this interface is dynamic rather than unidirectional: clinical signals shape sensing, sensing informs the Governance Cell, and the Governance Cell, in turn, influences ongoing sensing and interpretation. This interface defines the point at which clinical reality and governance meet. Its integrity is essential for enabling the assignment of responsibility and coordinated system response, as illustrated in Figure 2.

At the most fundamental level, governance depends on a clinical interface through which frontline information engages the system (Figure 1). This interface establishes the foundation for the broader governance structure that follows. This interface establishes the foundation for the broader governance structure that follows. Clinical Signals, arising from patient care, are bidirectionally linked with Sensing, representing the ongoing recognition and contextual interpretation of emerging concerns. Sensing is, in turn, bidirectionally coupled with the Governance Cell, which is anchored in the clinician–patient relationship and defines continuity as a central feature. This interface is where clinical reality and governance meet and must remain intact for the system to function effectively.

**FIGURE 2:**
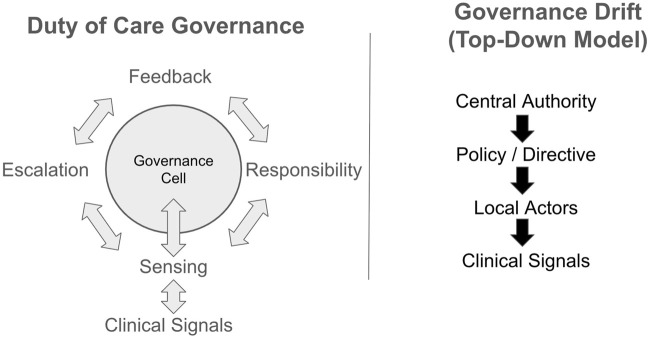
Contrasting governance architectures: Duty of Care Governance and Governance Drift. Legend: This figure contrasts two governance architectures and their implications for system responsiveness. On the left, Duty of Care Governance is organized around the Governance Cell, which is anchored in the clinician–patient relationship. Clinical Signals are engaged through Sensing, and this interface supports the assignment of Responsibility through coordinated governance processes. Escalation pathways allow concerns to move appropriately within the system, while Feedback ensures that information continues to circulate between the Governance Cell and frontline care. In this configuration, Sensing, Responsibility, Escalation, and Feedback are integrated, enabling Clinical Signals to inform governance in a timely, continuous manner. On the right, Governance Drift is depicted as a contrasting model in which authority flows in a predominantly top-down manner from central decision-making structures. In this configuration, Clinical Signals are weakly connected to governance processes, Sensing is attenuated, Escalation pathways are constrained, and Feedback is limited or absent. The Governance Cell is absent or functionally disconnected, and Responsibility becomes decoupled from frontline clinical reality. Together, these contrasting structures illustrate how the presence or absence of an integrated Governance Cell, along with effective Sensing, Responsibility, Escalation, and Feedback determine system responsiveness.

Building on this interface, Duty of Care Governance extends to a broader system in which the four governance functions, Sensing, Responsibility, Escalation, and Feedback, are integrated across institutional structures (Figure 2). Clinical Signals are incorporated into governance through Sensing, while Responsibility is defined and maintained at the clinician level. Escalation provides access to additional expertise without loss of primary decision authority, and Feedback enables continuous reassessment over time. Together, these elements form a recursive, bidirectional governance cycle that supports coordinated system response while remaining anchored in the clinician–patient relationship.

In contrast, Governance Drift describes a structural condition in which alignment between clinical reality and governance breaks down (Figure 2). In such configurations, authority becomes predominantly top-down, and the integration of Clinical Signals into decision-making is weakened. Sensing is attenuated, Escalation pathways are constrained, and Feedback is limited or absent. The Governance Cell is either absent or functionally disconnected, resulting in a progressive shift of decision authority away from the clinician–patient relationship and toward administrative control.

Duty of Care Governance can thus be defined as a relationally anchored governance model in which the clinician–patient relationship serves as the core unit of accountability, Clinical Signals function as the primary informational substrate, and system coherence is maintained through the integrated operation of Sensing, Responsibility, Escalation, and Feedback within a continuous, bidirectional governance structure.

## Metrics without moral proximity

The Duty of Care Governance framework centers authority at the site of accountability (the clinician–patient relationship). It is critical to address how existing governance tools can cause relational displacement, the process where efficiency-driven standardization and centralization displace the primary professional bond between the patient and clinician, a pattern aligned with evidence linking care continuity to improved outcomes (Starfield et al., 2005; Pereira Gray et al., 2018). This can contribute to fragmentation in care delivery (Yarnall et al., 2003), widening the gap between clinical decision-making and patient outcomes. Metric-based governance is a dual-edged tool in modern health systems. While it can improve transparency and reveal variation, it can also shape care delivery in ways that risk relational displacement (Beauvais et al., 2023; McEwen and Akil, 2020). While performance dashboards can improve transparency and reveal care variations, they do not inherently ensure that relational and patient-centered aspects of care are preserved (Beauvais et al., 2023). This requires a critical distinction between technical/safety compliance, such as infection control and data integrity, which serve a protective function, and administrative/managerial compliance, which focuses on billing targets and throughput optimization, informed by policy frameworks that differentiate quality and performance metrics (Centers for Medicare and Medicaid Services, 2024).

The ethical logic of care can be displaced when governance systems prioritize efficiency-driven metrics over the interpretive judgment required within clinical relationships, a dynamic reflected in evidence on clinician burnout and misalignment between professional values and system demands (Shanafelt et al., 2017). This creates a moral proximity gap as decision authority aggregates at administrative levels, distant from the specific consequences of individual patient care (Starfield et al., 2005). For clinicians, this misalignment often manifests as moral distress, where institutional mandates for standardized approaches conflict with professional values and obligations to individual patients, contributing to burnout and disengagement (Shanafelt et al., 2017).

It is essential to note that the goal of the Duty of Care Governance model is not to shift liability, as the institution typically bears the ultimate legal and operational Responsibility for adverse outcomes. Instead, the clinician’s Responsibility function ensures professional accountability for interpretive judgment, providing a mechanism for challenging procedural compliance when it threatens the patient’s best interest, supported by evidence on the central role of clinicians and relational continuity in care (Starfield et al., 2005). Within this framework, clinician accountability for interpretive judgment operates alongside institutional accountability for system design and safety. When clinical decisions deviate from standardized protocols, accountability is not removed but becomes traceable, enabling structured review, peer evaluation, and institutional learning rather than silent override or diffuse Responsibility. The model’s purpose is to ensure that institutional authority remains a faithful extension of the clinician–patient relationship, rather than a source of silent overrides, thereby preserving relational continuity (Pereira Gray et al., 2018). These dynamics become most visible when examined in the context of complex, longitudinal clinical conditions.

## Clinical stress test: Primary Ovarian Insufficiency

The structural tension between administrative scaling and clinical Responsibility becomes most visible when tested against complex, longitudinal conditions, where continuity and coordination of care are essential (Starfield et al., 2005). Primary Ovarian Insufficiency serves as a definitive stress test for governance, given its complex, multi-system, and longitudinal nature, which includes diverse etiologies spanning genetic, environmental, and idiopathic factors (Nelson, 2009; Gordon et al., 2017; Fink et al., 2018; Panay et al., 2024; An et al., 2025; Bakhsh, 2025). This complexity is further reflected in the need for timely diagnosis and access to accurate, comprehensible information, which are central to the experiences and needs of affected individuals (Alzubaidi et al., 2002). As a rare, multi-system disorder that unfolds over decades, Primary Ovarian Insufficiency is structurally unforgiving of Governance Drift when authority is separated from the site of care, given its reliance on longitudinal, coordinated clinical management (Nelson, 2009). Because clinical management of this condition intersects diverse specialties, including endocrinology, bone health, and mental health, and requires careful diagnostic evaluation across developmental stages, it requires sustained coordination across domains and highlights the need for a coherent relational center in care delivery (Covington et al., 2011; American College of Obstetricians and Gynecologists, 2018; Gaspari et al., 2023; Panay et al., 2024; Lumsden et al., 2025; Borahay et al., 2025).

To counter this potential for dissolution, this framework reconstructs the clinician–patient relationship as a primary Governance Cell, emphasizing its central role in sustaining longitudinal care (Pereira Gray et al., 2018). Within this cell, Sensing is operationalized through the patient’s and clinician’s interpretation of longitudinal Clinical Signals (such as fluctuating laboratory markers combined with the patient’s embodied experience of symptoms), reflecting a life-course approach to health and illness (Kuh et al., 2003; Harlow et al., 2012). For example, if a patient reports persistent, nonspecific fatigue (“the ouch”), the clinician interprets this, in the context of the patient’s full history, as a potential indicator of clinical depression, a recognized psychological consequence of Primary Ovarian Insufficiency (Davis et al., 2010; Schmidt et al., 2011; Covington et al., 2011). Responsibility remains identifiable, with the clinician serving as a named accountable individual for the patient’s health trajectory across her lifespan, reflecting the central role of primary care and continuity in care delivery (Starfield et al., 2005).

To support this cell without overriding its authority, the model utilizes transparent Escalation pathways. If the clinician orders a DEXA scan, they are doing so based on the increased risk of osteoporosis in patients with Primary Ovarian Insufficiency (a Clinical Signal) (Popat et al., 2014; Panay et al., 2024; Lumsden et al., 2025). If an automated system flags this scan as “non-standard” based only on the patient’s age, the clinician can use the clear Escalation pathway to bypass the algorithmic default. This allows them to justify the test based on their Responsibility and the patient’s longitudinal data. They integrate expert consultation without surrendering primary decision-making, preserving clinician-led coordination of care (Starfield et al., 2005). Continuous Feedback loops then ensure that treatment adjustments are based on this shared history of care rather than isolated data points, reinforcing relational continuity (Pereira Gray et al., 2018).

To reinforce this architecture, two conceptual anchors are proposed. A continuity steward (navigator) maintains longitudinal coherence by consolidating records and flagging potential “silent overrides” by automated systems. Furthermore, an organizational charter serves as a stabilizing constraint, codifying ethical commitments to patient decision primacy. Within this model, participatory design and patient engagement inform care processes and decision-making (Marzban et al., 2022). Clinical decision-making authority, however, remains anchored in the clinician–patient relationship. It is important to note that these roles are presented here conceptually to illustrate the application of Duty of Care Governance; the navigator and organizational charter are not currently implemented as standardized models for Primary Ovarian Insufficiency. The implications of this model extend beyond this clinical context, informing how health systems can be structured to align technological, organizational, and financial dimensions with the relational core of care.

## System-wide implications and financial imperative

The structural discipline addressed by the Duty of Care Governance model offers a blueprint for aligning the entire system, including its technology and financial strategy, emphasizing coordinated, relationship-based care (Starfield et al., 2005). The model addresses a shared problem across patient care and clinical investigation: the tendency for centralized management and digital monitoring to shift decision-making away from clinician judgment at the point of care (Grace et al., 2025). As automated tools and computer-guided systems increasingly shape care environments, their governance requires transparency to ensure safe and accountable use (Grace et al., 2025). These technologies need to function as a supportive infrastructure that preserves human understanding and provides clear Escalation lanes for clinicians to challenge automated defaults, reinforcing the central role of the clinician–patient relationship (Pereira Gray et al., 2018).

This structural stability also addresses a critical financial necessity. While health system management may sometimes focus narrowly on cost-cutting and increasing patient volume, this approach can generate downstream costs by undermining relational continuity and coordinated care, which are associated with improved outcomes (Starfield et al., 2005). The model argues that the true costs of misalignment manifest as compounding, often unrecognized burdens, including fragmented care and the increasing demands placed on patients to coordinate their own care (Yarnall et al., 2003). This emphasis on relational quality is reflected in existing policy structures. For example, the Centers for Medicare and Medicaid Services Hospital Value-Based Purchasing program incorporates patient experience as a measurable component of system performance, demonstrating that relational aspects of care are formally integrated into health system performance frameworks (Centers for Medicare and Medicaid Services, 2024).

While a full economic evaluation is beyond the scope of this manuscript, existing evidence linking continuity of care and clinician wellbeing to improved outcomes suggests that such misalignment carries measurable downstream costs. This framing is conceptual and intended to guide future economic evaluation. The requirements of this model can be viewed as smart spending to prevent future liabilities. These investments may mitigate risks such as staff turnover and adverse events (Shanafelt et al., 2017; Beauvais et al., 2023), rather than constituting unproductive bureaucratic spending that does not improve patient safety or system performance. This strategy contrasts these long-term financial liabilities with the value of sustained, long-term care and improved patient outcomes (Pereira Gray et al., 2018; Starfield et al., 2005), closely associated with patient trust.

Beyond its implications for patient care and clinician accountability, Duty of Care Governance may also be relevant to broader stakeholder alignment within health systems, including investors, contractors, administrative personnel, and suppliers. In this context, the model can be understood as an enterprise-level governance logic that serves as a unifying framework across clinical, operational, and financial domains. When governance structures maintain clear lines of responsibility and preserve the integrity of decision-making at the point of care, they reduce systemic ambiguity and the risk of downstream failure, consistent with principles described in high-reliability systems (Chassin and Loeb, 2013; Christianson et al., 2011; Weick and Sutcliffe, 2015). This clarity can support more predictable operational environments and reduce exposure to reputational and financial risk across the system. In this sense, governance models that sustain relational accountability may contribute not only to clinical outcomes but also to institutional stability and long-term value creation, as suggested by evidence linking continuity of care to improved outcomes and reduced system costs (Starfield et al., 2005; Pereira Gray et al., 2018; Bazemore et al., 2018; Barker et al., 2017).

## Conclusion

Modern health systems face a structural challenge: centralized authority can weaken the clinician’s duty of care to the patient, creating risks including erosion of the clinician–patient relationship and increased clinician burnout. This manuscript introduces Duty of Care Governance as a conceptual structural model intended to help realign institutional design with this foundational obligation. By anchoring Responsibility in the clinician–patient relationship and organizing governance around four interrelated functions, Sensing, Responsibility, Escalation, and Feedback, the model is designed to preserve the integrity of clinical judgment within complex systems through a coordinated governance structure linking Clinical Signals with institutional guidance.

The model further suggests that legitimacy depends on collaborative design, in which patients and clinicians co-author governing structures, such as an organizational charter, to reduce relational displacement and organizational inertia. This approach is intended to ensure that institutional authority remains connected to clinical experience while maintaining clear clinical accountability.

From a financial perspective, Duty of Care Governance may be understood as a strategic investment rather than an administrative overhead. By preventing misalignment, the model may help mitigate long-term liabilities, including clinician burnout, system inefficiencies, and patient burden, while potentially generating measurable value through improved outcomes and sustained trust. Ultimately, health systems that maintain structural discipline and keep authority centered in the clinician–patient relationship may be better positioned to scale without losing the ethical foundation of care. This model provides a conceptual framework that may inform efforts to achieve that balance.

## Data Availability

The original contributions presented in the study are included in the article/supplementary material, further inquiries can be directed to the corresponding author.

## References

[B1] Alzubaidi N. H. Chapin H. L. Vanderhoof V. H. Calis K. A. Nelson L. M. (2002). Meeting the needs of young women with secondary amenorrhea and spontaneous premature ovarian failure. Obstet. Gynecol. 99, 720–725. 10.1016/s0029-7844(02)01962-2 11978278

[B2] American College of Obstetricians and Gynecologists (2018). ACOG committee opinion no. 755: well-woman visit. Obstet. Gynecol. 132, e181–e186. 10.1097/AOG.0000000000002897 30247364

[B3] An L. Huang Y. Wang Y. Shen S. Luo X. Liang X. (2025). Assessment of ovarian dysfunction induced by environmental toxins: a systematic review. Front. Public Health 13, 1575418. 10.3389/fpubh.2025.1575418 40809751 PMC12343636

[B4] Bakhsh H. (2025). An evidence-based approach to the management of primary ovarian insufficiency in adolescents and young women. Life (Basel) 15, 1366. 10.3390/life15091366 41010308 PMC12471269

[B5] Barker I. Steventon A. Deeny S. R. (2017). Association between continuity of care in general practice and hospital admissions for ambulatory care sensitive conditions: cross sectional study of routinely collected, person level data. BMJ 356, j84. 10.1136/bmj.j84 28148478

[B6] Bazemore A. Petterson S. Peterson L. E. Bruno R. Chung Y. Phillips R. L. (2018). Higher primary care physician continuity is associated with lower costs and hospitalizations. Ann. Fam. Med. 16, 492–497. 10.1370/afm.2308 30420363 PMC6231930

[B34] Beauvais B. Kruse C. S. Ramamonjiarivelo Z. Pradhan R. Sen K. Fulton L. (2023). An exploratory analysis of the association between hospital labor costs and the quality of care. Risk Manag. Healthc. Policy 16, 1075–1091. 10.2147/RMHP.S410296 37342727 PMC10278947

[B7] Borahay M. A. Alperin M. Turok D. Coutifaris C. Santoro N. Burd I. (2025). Addressing the crisis in women’s health research: an American gynecological and obstetrical society statement. Am. J. Obstet. Gynecol. 233, 79–81. 10.1016/j.ajog.2025.05.002 40450415

[B37] Centers for Medicare & Medicaid Services (2024). Quality Payment Program. Centers for Medicare & Medicaid Services. Available online at: https://qpp.cms.gov/ (Accessed May 9, 2026).

[B8] Chassin M. R. Loeb J. M. (2013). High-reliability health care: getting there from here. Milbank Q. 91, 459–490. 10.1111/1468-0009.12023 24028696 PMC3790522

[B9] Christianson M. K. Sutcliffe K. M. Miller M. A. Iwashyna T. J. (2011). Becoming a high reliability organization. Crit. Care 15, 314. 10.1186/cc10360 22188677 PMC3388695

[B10] Covington S. N. Hillard P. J. Sterling E. W. Nelson L. M. , and Primar y Ovarian Insufficiency Recovery Group (2011). A family systems approach to primary ovarian insufficiency. J. Pediatr. Adolesc. Gynecol. 24, 137–141. 10.1016/j.jpag.2010.12.004 21269850 PMC3094722

[B11] Davis M. Ventura J. L. Wieners M. Covington S. N. Vanderhoof V. H. Ryan M. E. (2010). The psychosocial transition associated with spontaneous 46,XX primary ovarian insufficiency. Fertil. Steril. 93, 2321–2329. 10.1016/j.fertnstert.2008.12.122 19243752 PMC3013503

[B12] Fink D. A. Nelson L. M. Pyeritz R. Johnson J. Sherman S. L. Cohen Y. (2018). Fragile X associated primary ovarian insufficiency (FXPOI): case report and literature review. Front. Genet. 9, 529. 10.3389/fgene.2018.00529 30542367 PMC6278244

[B13] Freidson E. (1970). Profession of medicine: a study of the sociology of applied knowledge. Chicago, IL: University of Chicago Press.

[B14] Gaspari L. Paris F. Kalfa N. Sultan C. (2023). Primary amenorrhea in adolescents: approach to diagnosis and management. Endocrines 4, 536–547. 10.3390/endocrines4030038

[B15] Gordon C. M. Ackerman K. E. Berga S. L. Kaplan J. R. Mastorakos G. Misra M. (2017). Functional hypothalamic amenorrhea: an endocrine society clinical practice guideline. J. Clin. Endocrinol. Metab. 102, 1413–1439. 10.1210/jc.2017-00131 28368518

[B16] Grace B. Wise L. A. Nieroda M. Egbunike J. Usman N. O. (2025). Digital health technologies to transform women’s health innovation and inclusive research. BMJ 391, e085682. 10.1136/bmj-2025-085682 41073079 PMC12509992

[B17] Harlow S. D. Gass M. Hall J. E. Lobo R. Maki P. Rebar R. W. (2012). Executive summary of the stages of reproductive aging workshop +10. Menopause 19, 387–395. 10.1097/gme.0b013e31824d8f40 22343510 PMC3340903

[B18] Kuh D. Ben-Shlomo Y. Lynch J. Hallqvist J. Power C. (2003). Life course epidemiology. J. Epidemiol. Community Health 57, 778–783. 10.1136/jech.57.10.778 14573579 PMC1732305

[B19] Law.com (2026). Duty of care definition. Available online at: https://dictionary.law.com/Default.aspx?selected=599 (Accessed April 21, 2026).

[B20] Lumsden M. A. Dekkers O. M. Faubion S. S. Lindén Hirschberg A. Jayasena C. N. Lambrinoudaki I. (2025). European society of endocrinology guideline. Eur. J. Endocrinol. 193, G49–G81. 10.1093/ejendo/lvaf206 41082911

[B21] Marzban S. Najafi M. Agolli A. Ashrafi E. (2022). Impact of patient engagement on healthcare quality: a scoping review. J. Patient Exp. 9, 23743735221125439. 10.1177/23743735221125439 36134145 PMC9483965

[B22] McEwen B. S. Akil H. (2020). Revisiting the stress concept. J. Neurosci. 40, 12–21. 10.1523/JNEUROSCI.0733-19.2019 31896560 PMC6939488

[B33] Moore M.-L. Riddell D. Vocisano D. (2015). Scaling out, scaling up, scaling deep: strategies of non-profits in advancing systemic social innovation. J. Corp. Citiz. 2015 (58), 67–54. Available online at: https://www.jstor.org/stable/jcorpciti.58.67 .

[B23] Nelson L. M. (2009). Primary ovarian insufficiency. N. Engl. J. Med. 360, 606–614. 10.1056/NEJMcp0808697 19196677 PMC2762081

[B24] Nelson L. M. (2026). Trauma, coherence, and vigilance across scales. Front. Public Health 14, 1794313. 10.3389/fpubh.2026.1794313 41938922 PMC13047166

[B25] Panay N. Anderson R. A. Bennie A. Cedars M. Davies M. Ee C. (2024). Evidence-based guideline: premature ovarian insufficiency. Hum. Reprod. Open 2024, hoae065. 10.1093/hropen/hoae065 39660328 PMC11631070

[B26] Pereira Gray D. J. Sidaway-Lee K. White E. Thorne A. Evans P. H. (2018). Continuity of care. BMJ Open 8, e021161. 10.1136/bmjopen-2017-021161 29959146 PMC6042583

[B27] Popat V. B. Calis K. A. Kalantaridou S. N. Vanderhoof V. H. Koziol D. Troendle J. F. (2014). Bone mineral density in young women with primary ovarian insufficiency. J. Clin. Endocrinol. Metab. 99, 3418–3426. 10.1210/jc.2013-4145 24905063 PMC4154086

[B36] Shanafelt T. D. Dyrbye L. N. West C. P. (2017). DAddressing physician burnout: the way forward. JAMA 317, 901–902. 10.1001/jama.2017.0076 28196201

[B28] Scally G. Donaldson L. J. (1998). Clinical governance and the drive for quality improvement. BMJ 317, 61–65. 10.1136/bmj.317.7150.61 9651278 PMC1113460

[B35] Schmidt K.-H. Diestel S. (2011). Differential effects of decision latitude and control on the job demands-strain relationship: a cross-sectional survey study among elderly care nursing staff. Int. J. Nurs. Stud. 48, 307–317. 10.1016/j.ijnurstu.2010.04.003 20472236

[B29] Starfield B. Shi L. Macinko J. (2005). Contribution of primary care. Milbank Q. 83, 457–502. 10.1111/j.1468-0009.2005.00409.x 16202000 PMC2690145

[B30] Weick K. E. Sutcliffe K. M. (2015). Managing the unexpected: sustained performance in a complex world. 3rd Edn. Hoboken, NJ: Wiley.

[B31] Yarnall K. S. H. Pollak K. I. Østbye T. Krause K. M. Michener J. L. (2003). Is there enough time for prevention? Am. J. Public Health 93, 635–641. 10.2105/ajph.93.4.635 12660210 PMC1447803

